# Preferences for oral-fluid-based or blood-based HIV self-testing and provider-delivered testing: an observational study among different populations in Zimbabwe

**DOI:** 10.1186/s12879-023-08624-y

**Published:** 2023-10-17

**Authors:** Webster Mavhu, Memory Makamba, Karin Hatzold, Galven Maringwa, Albert Takaruza, Miriam Mutseta, Getrude Ncube, Frances M. Cowan, Euphemia L. Sibanda

**Affiliations:** 1 Centre for Sexual Health and HIV/AIDS Research (CeSHHAR) Zimbabwe, 4 Bath Road, Belgravia, Harare, Zimbabwe; 2 Department of International Public Health, Liverpool School of Tropical Medicine, Liverpool, UK; 3 Population Services International, Cape Town, South Africa; 4 Population Solutions for Health, Harare, Zimbabwe; 5 Ministry of Health and Child Care, Harare, Zimbabwe

**Keywords:** HIV self-testing, Female sex workers, Men, Observational study, Zimbabwe

## Abstract

**Background:**

There is limited data on client preferences for different HIV self-testing (HIVST) and provider-delivered testing options and associated factors. We explored client preferences for oral-fluid-based self-testing (OFBST), blood-based self-testing (BBST) and provider-delivered blood-based testing (PDBBT) among different populations.

**Methods:**

At clinics providing HIV testing services to general populations (1 urban, 1 rural clinic), men seeking voluntary medical male circumcision (VMMC, 1 clinic), and female sex workers (FSW, 1 clinic), clients had the option to test using OFBST, BBST or PDBBT. A pre-test questionnaire collected information on demographics and testing history. Two weeks after collecting a self-test kit, participants responded to a questionnaire. We used logistic regression to determine predictors of choices. We also conducted 20 in-depth interviews to contextualise quantitative findings.

**Results:**

May to June 2019, we recruited 1244 participants of whom 249 (20%), 251 (20%), 244 (20%) and 500 (40%) were attending urban general, rural, VMMC and FSW clinics, respectively. Half (*n* = 619, 50%) chose OFBST, 440 (35%) and 185 (15%) chose BBST and PDBBT, respectively. In multivariable analysis comparing those choosing HIVST (OFBST and BBST combined) versus not, those who had never married aOR 0.57 (95% CI 0.34–0.93) and those previously married aOR0.56 (0.34–0.93) were less likely versus married participants to choose HIVST. HIVST preference increased with education, aOR 2.00 (1.28–3.13), 2.55 (1.28–5.07), 2.76 (1.48–5.14) for ordinary, advanced and tertiary education, respectively versus none/primary education. HIVST preference decreased with age aOR 0.97 (0.96–0.99). Urban participants were more likely than rural ones to choose HIVST, aOR 9.77 (5.47–17.41), 3.38 (2.03–5.62) and 2.23 (1.38–3.61) for FSW, urban general and VMMC clients, respectively. Comparing those choosing OFBST with those choosing BBST, less literate participants were less likely to choose oral fluid tests, aOR 0.29 (0.09–0.92).

**Conclusions:**

Most testing clients opted for OFBST, followed by BBST and lastly, PDBBT. Those who self-assessed as less healthy were more likely to opt for PDBBT which likely facilitated linkage. Results show importance of continued provision of all strategies in order to meet needs of different populations, and may be useful to inform both HIVST kit stock projections and tailoring of HIVST programs to meet the needs of different populations.

## Background

Human Immunodeficiency Virus (HIV) testing continues to be an essential entry point into HIV prevention, treatment, care and support services [1–3]. Despite initiatives to increase access to, and uptake of, HIV testing such as provider-initiated HIV testing and counselling [4], community-based approaches [5] and partner notification [6], HIV testing gaps remain, and in many settings important population groups including men [7, 8] and key populations such as female sex workers [9, 10] continue to be missed.

Men traditionally access health care less than women likely exacerbated by masculine norms which equate help-seeking with being “weak” [11–14]. Latest global estimates show that compared with women, 1 million more men living with HIV do not know their status, 1.8 million know their status but are not on treatment, and 1.6 million are not virally suppressed [15]. Female sex workers (FSW) are disproportionally affected by HIV and despite the well-publicised global decline in HIV infections in general populations, both HIV incidence and prevalence among FSW in Africa remain unacceptably high [15, 16]. Further, FSW underutilise conventional HIV services due to barriers at multiple levels including: high levels of stigma, discrimination, exclusion, violence, cost, convenience and confidentiality [10, 16]. It is therefore critical to explore innovative ways to overcome barriers to testing uptake, particularly among hard to reach populations including men and FSW.

HIV self-testing (HIVST) has been recommended as one approach to increase HIV testing uptake [3, 17–19]. There is evidence that in sub-Saharan Africa, HIVST is safe [20, 21], feasible [22–24], acceptable [3, 25] and cost-effective [26, 27]. There is, however, currently limited data on preferences for different self-testing and provider-delivered testing options and associated factors. Available HIV testing modalities include oral-fluid-based self-testing (OFBST), blood-based self-testing (BBST) and provider-delivered blood-based testing (PDBBT). There are perceived advantages and disadvantages of each of these methods. For example, oral-fluid-based self-testing is widely viewed as simple, quick, painless and less invasive [10, 16], but characterised by lower sensitivity [24, 28]. Blood-based self-testing is perceived as more accurate [10] and having a higher sensitivity [24, 29], but more invasive and complex to perform. Provider-delivered testing often encompasses more counselling support and facilitates quicker linkage to post-test services [10] (confirmatory testing, voluntary medical male circumcision (VMMC), antiretroviral therapy, pre-exposure prophylaxis). Provider-delivered testing, however, may be less convenient and confidential than self-testing [30, 31].

Given the perceived advantages and disadvantages of the three HIV testing modalities, we conducted an observational study that explored client preferences for these among different populations (including men and FSW) in Zimbabwe. Findings will inform both HIVST kit stock projections and tailoring of HIVST programs to meet the needs of different populations.

## Methods

### Study setting

We conducted the study at four facilities (*n* = 3 urban; *n* = 1 rural) offering HIV testing services. The rural facility plus one urban provided these services to the general population, while the other two urban facilities offered services to men seeking VMMC and to FSW respectively.

### Study participants

Participants were eligible for the study if they were ≥ 18 years old, unaware of their HIV status and were using a mobile phone and willing to share their phone number for follow-up. Additionally, men recruited at the VMMC clinic needed to be willing to undergo VMMC; those already circumcised were excluded. We defined FSW as those attending a clinic for FSW.

### Study procedures

Clients seeking HIV testing services at any of the four facilities were given the option to test using one of three options: oral-fluid-based self-testing using the Oraquick self-test kit, blood-based self-testing – fingerstick blood-based testing using the Insti test or provider-delivered blood-based testing using fingerstick whole blood (first test from national testing algorithm using Determine, second test if positive or indeterminate using Chembio and tie breaker using Insti). Offer of the options was made by trained programme staff during group informational sessions (or to individuals if they came after these sessions) according to standard operating procedures, with description of what each testing option entailed.

A pre-test questionnaire collected information on demographics and testing history. Participants opting to self-test had a brief demonstration of how to use the HIV self-test kit. Two weeks after collecting the self-test kit, participants were contacted by phone to respond to a questionnaire on whether they had used the self-test, their self-testing experience, and any linkage to confirmatory testing. If the participant had a reactive self-test result and had not yet linked to confirmatory testing at the time of initial contact, a follow up call was made to encourage linkage three months later.

We also conducted 20 in-depth interviews (IDIs) with a subset of study participants to contextualise quantitative findings. Purposive sampling among participants who were offered the three testing options took into consideration age, sex, HIV testing method (i.e. whether selected OFBST, BBST or PDBBT) and residency (i.e., urban or rural) (Table 1). Participants were contacted approximately three months after the date they were tested by a provider (PDBBT) or collected a self-test. The in-depth interviews explored issues related to choice of testing method, self-testing process and experience and, any linkage to confirmatory testing. Interviews were informed by a topic guide and conducted by experienced and trained researchers in Shona, the participants’ language, and were audio recorded.

**Table 1:** In-depth interview participants’ demographic characteristics

Respondent ID	Age Median 30 years; IQR: 24–35	Female/Male	HIV testing method	Urban/Rural
01	24	Female	Blood-based self-testing	Urban
02	28	Male	Blood-based self-testing	Urban
03	20	Female	Blood-based self-testing	Urban
04	19	Female	Oral-fluid-based self-testing	Urban
05	46	Female	Provider-delivered blood-based testing	Urban
06	29	Male	Blood-based self-testing	Urban
07	30	Female	Oral-fluid-based self-testing	Urban
08	28	Male	Blood-based self-testing	Urban
09	35	Male	Oral-fluid-based self-testing	Rural
10	29	Female	Provider-delivered blood-based testing	Rural
11	34	Female	Provider-delivered blood-based testing	Rural
12	34	Female	Oral-fluid-based self-testing	Rural
13	36	Male	Oral-fluid-based self-testing	Urban
14	23	Male	Provider-delivered blood-based testing	Rural
15	47	Male	Blood-based self-testing	Urban
16	26	Female	Provider-delivered blood-based testing	Urban
17	35	Female	Oral-fluid-based self-testing	Urban
18	24	Male	Blood-based self-testing	Urban
19	42	Male	Provider-delivered blood-based testing	Urban
20	25	Male	Oral-fluid-based self-testing	Urban

### Sample size and power

We planned to recruit 1250 participants. Informed by a previous study [32], we assumed that 70% of the general population and VMMC clients and 50% of FSW would opt for self-testing, of whom 40–60% would choose blood-based self-testing. With these figures and assumptions, we would estimate the proportion of people choosing each option with precisions of 95% confidence intervals ranging from ± 7.3 to ± 8.6%.

### Data analysis

We conducted descriptive analyses to explore participant characteristics by place of recruitment. We calculated the proportion of participants choosing each of the testing options and compared differences by participants’ characteristics. We used Chi-square tests to test for the association between participants’ testing options and demographic variables. We generated a binary variable with categories of HIV self-testing (oral-fluid-based and blood-based self-testing combined) and provider-delivered blood-based testing.

Using univariable logistic regression, we determined the association between choosing self-testing and participants’ characteristics. We then conducted multivariable logistic regression, where we included variables at once in the model that were significant in the univariable logistic regression analysis at the 10% level of significance to obtain adjusted odds ratios (aOR). We also explored differences between participants who opted for oral-fluid-based and blood-based self-testing.

For the post-test survey data, we conducted a descriptive analysis. We presented proportions of participants opting for each testing modality, and, for self-testers, those who had used the test kits (disaggregated by choice of testing method (oral-fluid-based and blood-based self-testing)). We used Chi-square tests to determine association between choice of testing method and whether self-test kit was used. We used the same analysis to explore use of post-test services. Analyses were done with STATA v15.0 software (StatCorp, TX, USA).

For the qualitative data, analysis began during data collection where field notes were written for each in-depth interview, paying attention to emerging themes and how these would inform further exploration. After verbatim transcription and translation into English, detailed analytic summaries were written, with comparisons of themes within and across participants. Analytic summaries were used to compile a coding framework that was used by two researchers (MMk and WM) to code the data using NVivo 12 software (QSR International, Melbourne, Australia); care was taken to identify any additional emerging codes. Discrepancies were resolved through discussion (with ES) until consensus was reached. Codes were grouped and emerging themes were identified.

## Results

During May to June 2019, we recruited 1244 participants of whom 500 (40%), 251 (20%), 249 (20%) and 244 (20%) were attending FSW, rural, urban general and VMMC clinics, respectively. About two-thirds were female, 30% were < 25 years and a third were married. Two-thirds had ordinary level education and 10% had primary or no schooling, with almost all participants (99%) reporting being literate (could read a one-page letter or newspaper in English or Shona). Slightly over two-thirds (68%) did not receive a regular salary and the majority (87%) were Christian. Most (83%) rated their own health to be at least good and 92% had previously tested for HIV (Table 2).

**Table 2:** Observational study participants’ demographic characteristics

Participant characteristics	FSW *N* = 500 n (%)	Rural *N* = 251 n(%)	Urban general *N* = 249 n(%)	VMMC *N* = 244 n(%)	Total *N* = 1244 n(%)
Gender					
Male	0	82(33)	90(36)	244(100)	416(33)
Female	500(100)	169(67)	159(64)	0	828(67)
Age					
15–19 years	28(6)	28(11)	26(10)	28(11)	110(9)
20–24 years	107(21)	40(16)	43(17)	71(29)	261(21)
25–34 years	226(45)	68(27)	87(35)	80(33)	461(37)
35–44 years	111(22)	61(24)	61(25)	46(19)	279(22)
45 + years	28(6)	54(22)	32(13)	19(8)	133(11)
Marital status					
Married/cohabitating	8(2)	175(70)	117(47)	110(45)	410(33)
Never married	72(14)	42(17)	80(32)	116(48)	310(25)
Separated/widowed/divorced	420(84)	34(13)	52(21)	18(7)	524(42)
Level of education					
Primary/no schooling	72(14)	40(16)	6(2)	8(3)	126(10)
Form 1–4 (Ordinary level)	410(82)	142(57)	118(47)	156(64)	826(66)
Form 5–6 (Advanced level)	9(2)	26(10)	41(16)	43(18)	119(10)
Tertiary (College/university)	9(2)	43(17)	84(34)	37(15)	173(14)
Literacy (ability to read one- page letter/newspaper)					
Yes	494(99)	247(98)	246(99)	238(98)	1225(98)
No	6(1)	4(2)	3(1)	6(2)	19(2)
Receive regular salary					
Yes	106(21)	57(23)	119(48)	116(48)	398(32)
No	394(79)	194(77)	130(52)	128(52)	846(68)
Religion					
Christian	398(80)	235(94)	236(95)	207(85)	1076(87)
Muslim	6(1)	0	0	4(2)	10(1)
African traditional religion	11(2)	5(2)	5(2)	3(1)	24(2)
Other	5(1)	1(< 1)	0	4(2)	10(1)
No religion	80(16)	10(4)	8(3)	26(11)	124(10)
Own rating of health					
Very good	233(47)	75(30)	94(38)	139(57)	541(44)
Good	186(37)	102(41)	124(50)	82(34)	494(40)
Fair	73(15)	63(25)	25(10)	20(8)	181(15)
Poor	8(2)	11(4)	6(2)	2(1)	2(1)
Ever tested for HIV					
Yes	491(98)	225(90)	224(90)	208(85)	1148(92)
No	9(2)	26(10)	25(10)	36(15)	96(8)

### Testing preferences

Half of the participants (*n* = 619, 50%) opted for oral-fluid-based self-testing, while 440 (35%) and 185 (15%) opted for blood-based self-testing and provider-delivered blood-based testing, respectively. Table 3 shows participant preferences by socio-demographic characteristics.

**Table 3:** Participant preferences by socio-demographic characteristics

	OFBST *N* = 619 n (%)	BBST *N* = 440 n (%)	PDBBT *N* = 185 n(%)	Total
**Gender**				
Male	219(53)	136(33)	61(15)	416
Female	400(48)	304(37)	124(15)	828
**Population group**				
FSW	264(53)	201(40)	35(7)	500
Rural	101(40)	66(26)	84(33)	251
Urban general	128(51)	93(38)	28(11)	249
VMMC clients	126(52)	80(33)	38(16)	244
**Age (years)**	Mean (SD) 30.9 (9.3)	Mean (SD) 30.8 (9.3)	Mean (SD) 34.7 (12.8)	
15–19 years	51(46)	42(38)	17(15)	110
20–24 years	133(51)	96(37)	32(12)	261
25–34 years	241(52)	173(38)	47(10)	461
35–44 years	137(49)	90(32)	52(19)	279
45 + years	57(43)	39(29)	37(28)	133
**Marital status**				
Married/cohabitating	200(49)	128(31)	82(20)	410
Never married	159(51)	108(35)	43(14)	310
Separated/widowed/divorced	260(50)	204(39)	60(12)	524
**Level of education**				
Primary school/no schooling	52(41)	42(33)	32(25)	126
Form 1–4 (ordinary level)	408(48)	298(36)	120(15)	826
Form 5–6 (advanced level)	68(57)	37(31)	14(12)	119
Tertiary (college/university)	91(53)	63(36)	19(11)	173
**Literacy (ability to read one page letter/newspaper)**				
Yes	615(50)	430(35)	180(15)	1225
No	4(21)	10(53)	5(26)	19
**Receive regular salary**				
Yes	216(54)	131(33)	51(13)	398
No	403(48)	309(37)	134(16)	846
Total				
**Religion**				
Christian	528(49)	386(36)	162(15)	1076
Muslim	6(60)	4(40)	0	10
African traditional religion	12(50)	9(38)	3(13)	24
Other	6(60)	4(40)	0	10
No religion	67(54)	37(30)	20(16)	124
Total				
**Own rating of health**				
Very good	11(41)	10(37)	6(22)	27
Good	241(49)	178(36)	75(15)	494
Fair	296(55)	184(34)	61(11)	541
Poor	70(39)	68(38)	43(24)	181
Total				
**Ever tested for HIV**				
Yes	568(49)	413(36)	167(15)	1148
No	51(53)	27(28)	18(19)	96
Total				

Table 4 shows results of univariable and multivariable logistic regression analyses comparing participants who selected HIVST (oral-fluid-based and blood-based self-testing combined) with those who selected provider-delivered testing. In univariable analysis, several factors were associated with choice of either HIVST or provider-delivered testing including: population, age and marital status. In multivariable analysis where population group, age, marital status, education and own health rating were in the final model, FSW, urban general and VMMC participants were more likely to choose HIVST than rural participants with respective adjusted odds ratios (aOR) of 9.77 (5.47–17.41), 3.38 (2.03–5.62) and 2.23 (1.38–3.61). Preference for HIVST reduced with age, aOR for a one-year increase in age 0.97 (0.96–0.99). Participants who had never been married and those who were separated or divorced were less likely to choose HIVST, aOR 0.57 (0.34–0.93) and 0.56 (0.34–0.93), respectively. Preference for HIVST increased with education, score test for trend *p* < 0.001, where those who had attained tertiary education were more likely to choose HIVST compared to those who had primary or no education, aOR 3.50 (1.73–7.09). Participants who perceived their health to be either poor or fair were less likely to choose HIVST, aOR 0.85 (0.29–2.47) and aOR 0.62 (0.38–1.01), respectively compared to those who felt their health was very good, score test for trend *p* < 0.001.

**Table 4:** Crude and adjusted multivariable logistic regression models comparing factors associated with choosing HIVST over PDBBT

**Explanatory variables**	**Number (%)**	**N(%) choosing HIV self-tests**	**Odds Ratio (95% CI)**	***P*-value**	**Adjusted Odds Ratio (95% CI)**	***P*-value**
**Gender**				0.884		
Male (ref)	416(33)	355(85)	1			
Female	828(67)	704(85)	0.98(0.70–1.36)			
**Population group**				< 0.001		0.029
Rural (ref)	251(20)	167(67)	1		1	
FSW	500(40)	465(93)	6.68(4.34–10.3)		9.77(5.47–17.41)	
Urban general	249(20)	221(89)	3.97(2.47–6.37)		3.38(2.03–5.62)	
VMMC clients	244(20)	206(84)	2.73(1.77–4.21)		2.23(1.38–3.61)	
**Age (years)**		Mean (SD) 30.8 (9.3)	0.96(0.95–0.98)	< 0.001	0.97(0.96–0.99)	< 0.001
**Marital status**				< 0.001		0.041
Married/cohabitating (ref)	410(33)	328(80)	1		1	
Never married	310(25)	267(86)	1.55(1.04–2.32)		0.57(0.34–0.93)	
Separated/widowed/divorced	524(42)	464(89)	1.93(1.35–2.78)		0.56(0.34–0.93)	
**Education level**				< 0.001		0.005
Primary school/no school (ref)	126(10)	94(75)	1	< 0.001*	1	0.002*
Form 1–4 (ordinary level)	826(66)	706(85)	2.00(1.28–3.13)		1.55(0.92–2.61)	
Form 5–6 (advanced level)	119(10)	105(88)	2.55(1.28–5.07)		2.61(1.28–6.18)	
Tertiary (college/university)	173(14)	154(89)	2.76(1.48–5.14)		3.90(1.58–6.44)	
**Literate (able to read or write)**				0.167		
Yes (ref)	1225(98)	1045(85)	1			
No	19(2)	14(74)	0.48(0.17–1.36)			
**Receive regular salary**				0.163		
Yes (ref)	398(32)	347(87)	1			
No	846(68)	712(84)	0.78(0.55–1.10)			
**Religion**				0.889		
Christian (ref)	1076(86)	914(85)	1			
Muslim	10(1)	10(100)	1			
African traditional religion	24(2)	21(87)	1.24(0.37–4.21)			
Other	10(1)	10(100)	1			
No religion	124(10)	104(86)	0.92(0.56–1.53)			
**Own rating of health**				< 0.001		0.002
Very good (ref)	541(43)	480(89)	1	0.759*	1	< 0.001*
Good	494(40)	419(85)	0.71(0.49–1.02)		0.82(0.55–1.20)	
Fair	181(15)	138(76)	0.41(0.26–0.63)		0.62(0.38–1.00)	
Poor	27(2)	21(78)	0.44(0.17–1.15)		0.87(0.30–2.52)	
**Ever tested for HIV**				0.268		
Yes (ref)	1148(92)	981(85)	1			
No	96(8)	78(81)	0.74(0.43–1.26)			

^*^Level of education and own health rating: *p*-value for score test for trend of odds

Table 5 shows analysis of factors associated with choosing oral-fluid-based self-testing among participants who opted for the two self-testing options. Participants who reported being illiterate were less likely to choose oral-fluid-based self-testing, aOR 0.29 (0.09–0.92), *p* = 0.036. There was a trend towards less preference for oral-fluid-based self-testing among participants who did not receive a regular salary, aOR 0.80 (0.61–1.04) *p* = 0.091.

**Table 5:** Crude and adjusted multivariable logistic regression models comparing factors associated with choosing OFBST over BBST

Explanatory variables	Number (%)	N(%) choosing OFBST	Odds Ratio (95% CI)	*p*-value	Adjusted Odds Ratio (95% CI)	*P*-value
**Gender**				0.129		
Male (ref)	355(34)	219(62)	1			
Female	704(66)	400(57)	0.81(0.63–1.06)			
**Population group**				0.688		
Rural (ref)	167(16)	101(60)	1			
FSW	465(44)	264(57)	0.86(0.60–1.23)			
Urban general	221(21)	128(58)	0.90(0.60–1.35)			
VMMC clients	206(19)	126(61)	1.03(0.68–1.56)			
**Age (years)**		Mean (SD) 30.9 (12.8)	1.00(0.99–1.01)	0.844		
**Marital status**				0.348		
Married/cohabitating (ref)	328(31)	200(61)	1			
Never married	267(25)	159(60)	0.94(0.68–1.32)			
Separated/widowed/divorced	464(44)	260(56)	0.82(0.61–1.09)			
**Education level**				0.516		
Primary school/no school (ref)	94(8)	52(55)	1	0.383*		
Form 1–4 (ordinary level)	706(67)	408(58)	1.11(0.72–1.71)			
Form 5–6 (advanced level)	105(10)	68(65)	1.48(0.84–2.63)			
Tertiary (college/university)	154(15)	91(59)	1.17(0.69–1.96)			
**Literate (able to read/write)**				0.032		0.036
Yes (ref)	1045(99)	615(59)	1		1	
No	49(1)	4(29)	0.28(0.09–0.90)		0.29(0.09–0.92)	
**Receive regular salary**				0.080		0.091
Yes (ref)	347(33)	216(62)	1		1	
No	712(67)	403(57)	0.79(0.61–1.03)		0.80(0.61–1.04)	
**Religion**				0.779		
Christian (ref)	914(86)	528(58)	1			
Muslim	10(1)	6(60)	1.10(0.31–3.91)			
African traditional religion	21(2)	12(57)	0.97(0.41–2.34)			
Other	10(1)	6(60)	1.10(0.31–3.91)			
No religion	104(10)	67(64)	1.32(0.87–2.02)			
**Own rating of health**				0.118		
Very good (ref)	480(45)	296(62)	1	0.433*		
Good	419(40)	241(58)	0.84(0.64–1.10)			
Fair	138(13)	70(51)	0.64(0.44–0.94)			
Poor	21(2)	11(52)	0.68(0.28–1.64)			
**Ever tested for HIV**				0.198		
Yes (ref)	981(93)	568(58)	1			
No	78(7)	51(65)	1.37(0.85–2.23)			

^*^Level of education and own health rating: *p*-value for score test for trend of odds

### Post-test survey findings

In total, 1059 participants opted for HIVST (oral-fluid-based and blood-based self-testing), of whom 535 (51%) responded to the telephone questionnaire. Response rate was lowest among rural participants (58/167, 35%) owing to phone network connectivity challenges.

Of the 535 reached, 303 (57%) opted for oral-fluid-based self-testing and 232 (43%) for blood-based self-testing. Table 6 shows reasons for choosing each HIVST option. The most popular reason (35%) among those choosing oral-fluid-based self-testing was desire to try a new testing method, closely followed by belief that it would be easier to use (33%) and that there was no associated pain (28%). Among those choosing blood-based self-testing, the majority (71%) thought it was a more accurate test and 24% felt it would be easier to use.

**Table 6:** Reasons for opting for self-testing – post-test survey

**Reasons for choosing oral-fluid-based self-testing**	***N* = 294** **n(%)**
I wanted to try a new testing method	104 (35)
I felt it would be easier to use	97 (33)
There is no pain with oral fluid test	81 (28)
I felt this test would give a more accurate result	5 (2)
Other	7 (2)
**Reasons for choosing blood-based self-testing**	***N* = 227** n(%)
I felt it would be easier to use	54 (24)
I did not trust an oral fluid test	4 (2)
I felt this test would give a more accurate result	161 (71)
Other	8 (4)

Nearly all 299/303 (99%) and 224/232 (97%) of those that opted for oral-fluid-based self-testing and blood-based self-testing, respectively reportedly used the self-test kit. Of the 299 that reportedly used the oral-fluid-based self-test kit, 21 (7%) had a reactive result and 271 (91%) were HIV-negative while 5 (2%) had invalid results. Two (1%) did not wish to answer. Of the 224 that reportedly used the blood-based self-test kit, 12 (5%) had a reactive result and 193 (86%) were HIV-negative while 16 (7%) had invalid results. Three (1%) did not wish to answer.

### Qualitative findings

Qualitative data suggested that overall, participants preferred self-testing to provider-delivered testing for various reasons including relative flexibility and convenience. Men especially appreciated HIVST’s comparative advantages in relation to opportunity cost. *‘…When you seek services from a provider, you are given a lot of instructions …You are told to wait over there and so forth …Now, the issue of time, these days most of us are self-employed…’* (male IDI 20). FSW felt that HIVST would enable them to avert the discriminatory treatment they often experienced when accessing HIV testing services. *‘Nurses are very “rough” to us sex workers and so it will really help if we are able to test ourselves and not go to them every time’* (female IDI 4).

Discussions suggested that participants preferred oral-fluid-based self-testing to blood-based self-testing because they perceived it to be less invasive and painless, corroborating telephone survey findings. *‘I am one person who doesn’t like getting pricked. That is why I chose this other method of inserting something in my mouth [swabbing]*’ (male, IDI 13). Others chose oral-fluid-based self-testing simply because it was a new method. *‘I chose to test myself using the “mouth method” because previously, I had tested using blood and so I wanted to try the new method’* (male IDI 9). However, doubt over oral-fluid-based self-testing’s accuracy was a recurrent theme. Further probing revealed that this was largely because people could not understand how oral fluids could be tested for HIV when they had repeatedly learned that HIV cannot be transmitted through saliva. ‘*Isn’t it we have always been told that HIV cannot be transmitted through saliva? Why are they now saying saliva can show whether or not one has HIV?”* (female, IDI 11). Another participant also queried, *‘How is it possible that HIV can be found in saliva? We have always been “taught” that kissing does not transmit HIV. That is why I chose the one that involves blood’* (male, IDI 8).

Participants felt that compared to self-testing, provider-delivered testing likely resulted in more people testing. Participants described how hesitancy had resulted in some self-testing delays. *‘I waited for four days “asking myself” whether or not I should use the test kit”* (male, IDI 2). Additionally, they felt that provider-delivered testing facilitated quicker access to post-test services. As one participant put it, *‘The advantage of being tested by a “nurse” is that if she finds you positive [HIV], she will enrol you on the “programme” [colloquial term for antiretroviral therapy] there and then. There will be no delays…’* (female, IDI 5). Of note, among those reporting a reactive result, the two most cited reasons for not seeking confirmatory testing were not perceiving it as necessary and a lack of time/opportunity/money to attend.

## Discussion

We conducted an observational study to explore client preferences for three HIV testing modalities among different populations including men and FSW, population groups with recognised sup-optimal uptake of HIV services [7, 16, 30, 33]. Overall, we found that participants preferred oral-fluid-based self-testing, followed by blood-based self-testing and lastly, provider-delivered blood-based testing. A separate study conducted in Malawi also found a similar trend, with 60%, 38% and 2% opting for oral-fluid-based self-testing, blood-based self-testing and provider-delivered blood-based testing, respectively (O'Reilly et al., unpublished observations). Collectively, these findings can inform HIV self-testing (HIVST) kit stock projections.

This is one of the first programmatic evaluations of preferences for HIV testing among different population groups. When given a choice between self-testing and provider-delivered testing, 85% will choose self-testing, although this varied by population, with rural populations least likely to opt for self-testing. Overall younger, better educated, urban and sex worker populations were more likely to opt for HIVST. These findings have implications for the implementation of HIV testing services. It appears that increasing choice of testing modality increases acceptability and uptake of testing [34], and that different HIV testing approaches are preferred by different groups in different situations. HIVST may be particularly important for FSW, who often face high levels of stigma and discrimination, including from health providers [10, 35, 36], and need to test more regularly because of their heightened risk. HIVST may also be important for men who often have concerns about privacy, self-determination (control), convenience and flexibility [11, 37].

Rural participants were less likely than urban ones to choose HIVST. This may be related to their self-efficacy to self-test, and is in line with accuracy studies which showed that they were less able to produce accurate results [38]. Unsurprisingly, younger age and education were associated with preference for HIVST, also likely linked to self-efficacy and desire to embrace technological advances. Participants who felt that their health was poor or fair were less likely to choose HIVST, which could be driven by their need to get immediate provider attention and help should they test HIV-positive. This self-triage process has also been found in programmatic settings, where those opting for provider-delivered testing were more likely to test positive compared to those opting for self-testing [7]—a straightforward way of ensuring that those who need support from health care workers get it, while those in less urgent need use HIVST.

As found previously [10], some participants expressed doubts over the accuracy of oral-fluid-based self-testing with some preferring blood-based self-testing instead. Whilst the failure to appreciate that the oral-fluid-based self-testing that was used in the study detects antibodies rather than infectious virus is understandable, programmes promoting HIVST need to explain its diagnostic accuracy [39] and alleviate the doubts. Better understanding has the potential to further enhance acceptability and uptake of oral-fluid-based self-testing. In this study, we found that when given a choice, 15% will still choose provider-delivered testing over HIVST and that in rural, less educated populations this was over 40%. In addition, qualitative data suggested that provider-delivered testing facilitates easier linkage to services, particularly important for those at high risk of HIV transmission or acquisition (and in need of prevention), underlining the need to offer all testing modalities.

A strength of this observational study is that it explored preferences for a number of HIV testing modalities and among different populations thereby providing a range of perspectives. Whilst quantitative methods enabled us to quantify preferences, the qualitative enquiry enabled us to have an appreciation of factors behind the preferences, highlighting the value of mixed methods research [40].

A potential limitation is that we had a relatively large number of urban FSW whose perspectives may not represent those of their rural counterparts. Another limitation of the study is that although owning a mobile phone was a prerequisite for participating in the study, it was difficult to get hold of study participants to administer the post-test questionnaire. At 51%, the response rate was acceptable but still low to produce non-biased estimates of the self-testing experience. Also, we did not collect information on linkage to post-test services. Although linkage to post-test services for individuals who test away from health facilities may not be as immediate as when both testing and linkage are offered at the facility, there is evidence from our previous work that the offer of community-based HIV self-testing is associated with a significant increase in uptake of HIV treatment at nearby clinics [41]. Finally, many factors may motivate choice and preference. Therefore, choice at one point in time does not necessarily reflect overall preference, especially among those who have not been exposed to all options available.

## Conclusions

This observational study enabled us to explore, quantify and rank preferences for HIV testing modalities including by population groups. The most preferred testing option was oral fluid self-tests, followed by blood-based self-tests and lastly, provider-delivered tests. Results show importance of continued provision of all strategies in order to meet needs of different populations. Study findings will be important in ensuring the scale-up of HIVST in sub-Saharan Africa is tailored to meet the needs of different populations. Study findings are relevant to all programmes, ministries and implementers involved in scaling up HIVST.

## Data Availability

The datasets used and/or analysed during the current study are available from the corresponding author on reasonable request.

## Abbreviations

AOR: Adjusted odds ratio
BBST: Blood-based self-testing
CI: Confidence Interval
FSW: Female sex workers
HIV: Human Immunodeficiency Virus
HIVST: HIV self-testing
OFBST: Oral-fluid-based self-testing
PDBBT: Provider-delivered blood-based testing
VMMC: Voluntary medical male circumcision
